# Contrapositive logic suggests space radiation not having a strong impact on mortality of US astronauts and Soviet and Russian cosmonauts

**DOI:** 10.1038/s41598-019-44858-0

**Published:** 2019-07-04

**Authors:** Robert J. Reynolds, Igor V. Bukhtiyarov, Galina I. Tikhonova, Steven M. Day, Igor B. Ushakov, Tatyana Y. U. Gorchakova

**Affiliations:** 1 Mortality Research & Consulting, Inc., City of Industry, CA USA; 2 Federal State Budgetary Scientific Institution Izmerov Research Institute of Occupational Health, 31, Prospect Budennogo, Moscow, 105275 Russian Federation; 3 Russian State Research Center – Burnasyan Federal Medical Biophysical Center of Federal Medical Biological Agency, 46 Zhivopisnaya Street, 123182 Moscow, Russian Federation

**Keywords:** Epidemiology, Cardiology, Cancer epidemiology

## Abstract

Space travelers are exposed to unique forms of ionizing radiation that pose potentially serious health hazards. Prior analyses have attempted to quantify excess mortality risk for astronauts exposed to space radiation, but low statistical power has frustrated inferences. If exposure to deep space radiation were causally linked to deaths due to two particular causes, e.g., cancer and cardiovascular disease, then those cause-specific deaths would not be statistically independent. In this case, a Kaplan-Meier survival curve for a specific cause that treats deaths due to competing causes as uninformative censored events would result in biased estimates of survival probabilities. Here we look for evidence of a deleterious effect of historical exposure to space radiation by assessing whether or not there is evidence for such bias in Kaplan-Meier estimates of survival probabilities for cardiovascular disease and cancer. Evidence of such bias may implicate space radiation as a common causal link to these two disease processes. An absence of such evidence would be evidence that no such common causal link to radiation exposure during space travel exists. We found that survival estimates from the Kaplan-Meier curves were largely congruent with those of competing risk methods, suggesting that if ionizing radiation is impacting the risk of death due to cancer and cardiovascular disease, the effect is not dramatic.

## Introduction

From the earliest days of manned space exploration, the National Aeronautics and Space Administration (NASA) and the Soviet Space Program alike were concerned about exposure to ionizing radiation while in outer space. The concern is well-founded: being in outer space exposes astronauts and cosmonauts to several unique forms of ionizing radiation, which, in sufficient doses, are presumed to cause myriad health effects^1,2^.

Mechanisms by which exposure to ionizing radiation can increase the risk of mortality from cancers are well-known, and evidence is emerging regarding the role of ionizing radiation in the genesis of cardiovascular disease (CVD)^3–5^. So far, investigations into astronaut mortality have demonstrated no discernable excess mortality risk from cancer or CVD for astronauts in comparison to either the general population or professional athletes^6,7^. Explicit investigations of radiation exposure and cancer incidence in the astronaut cohort have similarly failed to detect any dose-response relationship^5,8^. However, limitations in interpretation due to low statistical power leave open the possibility of undetectably low levels of increased risk in this small sample, creating a need for the application of alternative epistemological approaches^5,8^.

If two potentially life-threatening processes (of disease, or lifestyle) share a common underlying cause, deaths due to those causes cannot be statistically independent events. In this situation cause-specific survival curves computed for each of the two causes under the assumption that any death due to the other cause is a case of uninformative censoring will provide biased estimates of (cause-specific) survival probabilities^9,10^.

In extreme cases this bias can lead to estimated total probabilities of death (i.e., sums of probabilities of death due to a number of separate possible causes) greater than 1.0^8^. To correct for bias resulting from such an erroneous assumption of uninformative censoring, methods of analysis that account for competing risks in the calculation of cumulative incidence can be used. Survival curves computed using such methods will necessarily be more optimistic than traditional Kaplan-Meier (K-M) estimates, yet if competing risks are independent (or at least not strongly dependent) the assumption of uninformative censoring will not be grossly wrong, and the differences in survival curves will be negligible^10^. On the other hand, strong enough dependence will create competing risk survival curves that are markedly different from naïve K-M curves (those that treat competing risks as mechanisms of uninformative censoring). Thus, these properties of survival curves for competing risks can be used to look for the presence of common causes between cause-of-death groups using *contrapositive reasoning*.

A contrapositive statement is one that refutes both the subject and the predicate of a conditional logical statement. Such a conditional statement takes the form of: *if A then B*. The contrapositive of this is *Not-B, therefore Not-A*. In this specific case, our logical statement is: *if astronauts are at increased risk of death from CVD and cancer due to a common underlying cause of ionizing radiation, then naïve cause-specific survival curves for each cause will be biased*. The contrapositive to this is: *If no bias is present in the naïve cause-specific survival curves, then there is no common underlying cause*. In this study we use this contrapositive reasoning to look for evidence of the possibility that doses of space radiation to date have increased mortality from CVD and cancer among US astronauts and Soviet and Russian cosmonauts.

## Results

### Characteristics of study populations

Table 1 presents actuarial and demographic characteristics of the study cohorts. The study population included 301 astronauts and 117 cosmonauts. There were just under 7,300 person-yrs of accrued follow-up time for astronauts and 3,000 for cosmonauts, with an average length of follow-up of 24 yrs per astronaut and 25 yrs per cosmonaut. There were 53 astronaut deaths during the study period and 36 cosmonaut deaths. For astronauts, the most common category of cause of death overall comprised external causes (including aircraft, spacecraft, and automobile accidents), accounting for 38% of the 53 astronaut deaths to date. The next most frequent cause group was cancer, accounting for 30% of the deaths so far. Other and unknown natural causes and CVD were less frequent, each accounting for approximately 15% of deaths. Among cosmonauts, the most common cause of death was CVD, with 50% of cosmonauts dying of this cause. Cancer was the next most common with 28%. External causes were responsible for a smaller proportion of deaths among cosmonauts in comparison to astronauts, accounting for 17% of cosmonaut deaths.

**Table 1:** Astronaut and cosmonaut group characteristics.

	Astronauts	Cosmonauts
N	301	117
Age at first flight, mean (sd)	40.7 (4.7)	38.3 (5.1)
**Years of follow-up**		
Total	7,295.8	2,966.8
Mean (sd)	24.2 (12.4)	25.4 (14.1)
Maximum	54.8	54.9
**Deaths, n (%)**		
All causes	53 (100.0)	36 (100.0)
Cancer	16 (30.2)	10 (28.0)
CVD	8 (15.1)	18 (50.0)
Other natural	6 (11.3)	2 (6.0)
External	20 (37.7)	6 (17.0)
Unknown	3 (5.7)	0 (0.0)

### Astronaut survival

**Figure 1:**
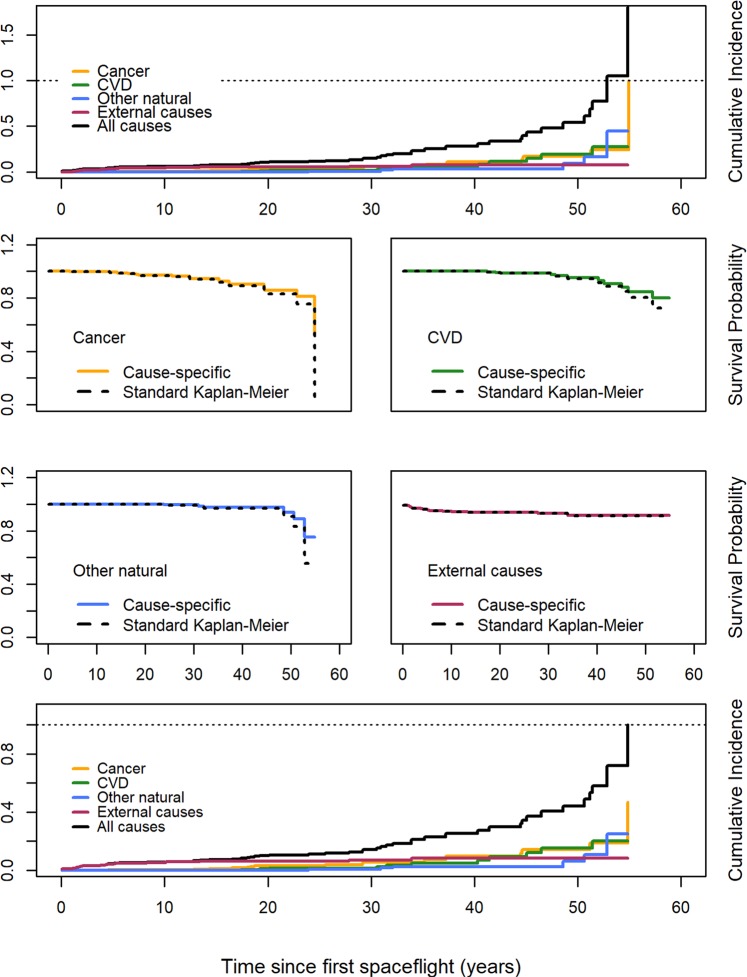
Cumulative incidence and survival curves for specific causes of death among US astronauts, 1959–2018. The upper panel shows the cumulative incidence from multiple causes when computed from naïve K-M survival estimates. The four middle panels provide naïve K-M and properly computed competing risk survival curves by cause. The bottom panel shows cumulative incidence curves by cause derived from competing risk survival curves.

Figure 1 shows the results of cumulative incidence (of mortality) and survival plots for US astronauts. The topmost plot shows cumulative incidence curves for each cause of death individually (by color) and for all causes combined (in black), computed using standard K-M methods with deaths due to competing causes treated as censored observations for each curve. The estimated probability of death by all-causes, computed here by adding the (naïve, K-M) estimates at each point, exceeds 1.7 at approximately 55 years. This is driven largely by the jump to 1.0 in the cumulative probability of death from cancer at that time. That the probability of death due to any one cause could reach 1.0, and that the sum of the probabilities of death due to each of the individual causes could exceed 1.0, indicate that bias is present in the naïve estimates of cause-specific survival probabilities.

The four plots in the middle of the panel show each properly computed cause-specific survival curve (properly accounting for competing causes of death) overlaid with the corresponding naïve K-M curve. As expected, the survival curve estimated by naïve K-M in all cases tracks lower than that estimated by competing-risk curves. However, the degree to which the curves diverge varies by cause. External causes show almost no divergence between the two curves while the plots for cancer, CVD, and other natural causes show divergence starting at approximately 40–45 years of follow-up. Cancer shows the largest divergence, with the competing risk survival estimate of 53% at 55 years instead of the K-M estimate of 0%. For CVD the difference between the K-M and competing risks curves was 7% after 55 yrs of follow-up, while other natural causes had a difference of 18% at 55 yrs.

Table 2 gives the areas under the curves (AUCs) for the curves in the 4 cause-specific plots of Fig. 1. Just as the competing risk curves give more optimistic survival estimates than the K-M curves, the AUCs for the competing risk curves are greater than those of the corresponding K-M curves. In spite of some of the large differences in end-of-period survival noted above, the differences in AUC were slight: the naïve K-M AUCs were all within 2% of the competing risk AUCs and the largest difference was still less than 1 person-year. The smallest ratio (indicative of the largest difference) was for other natural and unknown causes, with a ratio of 0.985 and a difference of 0.81 person-years. The largest ratio (smallest difference in AUC) was in external causes, with a ratio of 0.999 and a difference of 0.06 person-years.

**Table 2:** AUCs, AUC differences, and AUC ratios by cause of death category for K-M and competing risk survival curves for astronauts.

Cause of Death	AUC		Difference	Ratio
	K-M	CR		
Cancer	51.06	51.58	0.52	0.990
CVD	52.64	53.14	0.50	0.991
Other natural	51.79	52.60	0.81	0.985
External	51.15	51.21	0.06	0.999

The analyses in Fig. 1 and Table 2 have assumed that the three astronaut deaths due to unknown causes were due to other natural causes, rather than cancer, CVD, or external causes. We conducted separate analyses to examine the effect of adding those three deaths to the cancer or CVD category. When the three deaths from unknown causes were assumed to be cancer deaths, the survival estimate from the competing risk curve was 47% at 55 years, and the ratio of AUCs was reduced to 0.988. When instead we considered the unknown deaths to be from CVD, the competing-risk survival at 55 yrs was 80%, and the ratio of AUCs was 0.987. In both these scenarios, the survival for other natural causes increases to 75% at 55 yrs, and the ratio of AUCs increases slightly to 0.988.

The bottom-most plot in Fig. 1 shows all of the corrected cumulative incidence curves. As in the topmost plot, the black line is the sum of the individual cumulative incidence values at each point. In this plot the black line hits a maximum of 1.0 at approximately 55 yrs, demonstrating that the biases in the individual cause curves have indeed been corrected.

### Cosmonaut survival

**Figure 2:**
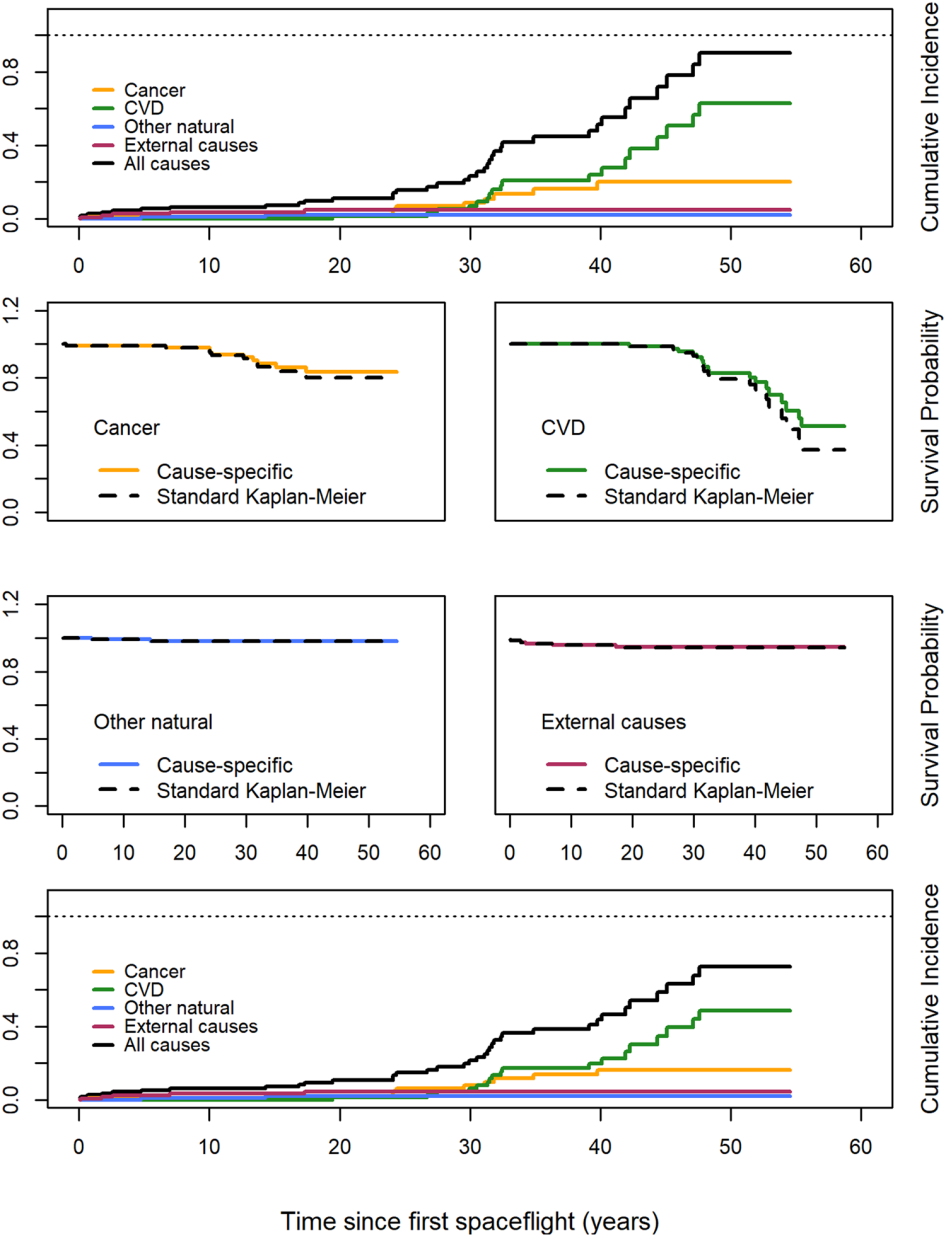
Cumulative incidence and survival curves for specific causes of death among Soviet and Russian cosmonauts, 1960–2017. The upper panel shows the cumulative incidence from multiple causes when computed from naïve K-M survival estimates. The four middle panels provide naïve K-M and properly computed competing risk survival curves by cause. The bottom panel shows cumulative incidence curves by cause derived from competing risk survival curves.

Figure 2 shows the same set of cumulative incidence and survival plots as in Fig. 1, in this case for cosmonauts. The all-cause cumulative incidence curve in the topmost plot shows no apparent bias in the cosmonaut cohort, as the curve does not exceed 1.0 at any point in the 55-yr follow-up. In the bottom-most plot, the all-cause cumulative incidence at 55 years of follow-up is 73%, compared to 90% in the original, biased version displayed in the top-most plot (black lines).

For both the other natural causes of death group and external causes of death group, the curves show almost no divergence across the follow-up period, with less than 1% difference in the curves for these two causes after 55 yrs of follow-up. However, there was approximately 3.5% divergence between the K-M and competing risks curves for cancer, and 14% divergence in the curves for CVD.

Table 3 contains the AUCs for the cause-specific survival curves of Fig. 2. Just as with astronauts, most K-M AUCs were within 1–2% of the competing risk AUCs with AUC differences of less than 1 person-year. The notable exception is CVD, where the K-M curve provided an AUC that was approximately 4% smaller than that of the competing risk curve. This equated to an AUC difference of 1.96 person-years.

**Table 3:** AUCs, AUC differences, and AUC ratios by cause of death category for K-M and competing risk survival curves for cosmonauts.

Cause of Death	AUC		Difference	Ratio
	K-M	CR		
Cancer	49.48	50.29	0.81	0.984
CVD	44.99	46.95	1.96	0.958
Other nat/unk	53.64	53.68	0.04	0.999
External	51.80	51.84	0.04	0.999

## Discussion

For US astronauts the naïve cumulative incidence function exceeded 1.0 at approximately 53 years of follow-up, but was corrected to be 0.72 at 53 years using competing risk methodology (Fig. 1). This is evidence of bias in the survival estimates using the naïve K-M method. This was driven largely by the cumulative incidence for cancer, which reached 1.0 at 55 years. However, in spite of this bias, we find that the ratios of the AUCs for all causes among astronauts were high, all at 0.985 or above. Similarly, the difference in the life-years lived under the various scenarios were small, less than 1 person-year in all cases. These figures suggest that even with late-period volatility in the survival estimate from the various causes, the naïve K-M survival curves are not markedly different from the competing risk curves overall. Thus, we conclude that the results presented here do not show evidence of strong interference in the survival curves for any of the natural-cause groups (cancer, CVD, other/unknown) among US astronauts. In keeping with the contrapositive reasoning employed here, we therefore conclude there is no common cause for death by cancer and death by CVD.

The patterns of survival in individual causes for cosmonauts were slightly different from those of astronauts, yet the overall results were similar. Cosmonaut survival curves did not show *prima facie* evidence of strong interference in Fig. 2, as the cumulative incidence curve did not exceed 1.0 during the follow-up period. The differences in survival between naïve K-M estimates and competing risk estimates were greatest for cancer and CVD, and nearly zero for other natural causes and external causes. The differences in the K-M and competing risk curves were most apparent in CVD, where the curves show clear and marked divergence starting at approximately 30 years of follow-up. This translated to the largest overall difference in survival curves among cosmonauts, with a 4.2% difference in the AUCs for CVD. While the largest of any difference in curves for cosmonauts or astronauts, and consistent with the hypothesis of space radiation as a common cause to both, the differences in the AUCs are still not large enough that we consider it evidence of strong interference, and thus do not consider it evidence of a common cause acting on both cancer and CVD mortality.

That there was virtually no bias in survival curves for external causes for astronauts or cosmonauts is unsurprising, as prior research has demonstrated no interference between external-cause survival and natural-cause survival in either cohort^11^. Externally-caused deaths have been overwhelmingly accidental for both astronauts and cosmonauts, and as such, the risk of death from these causes is comparatively low across the lifespan, with the result that accidental death does not interfere with estimates of survival from any natural causes. The lack of interference from the “other natural causes” group is also unsurprising, as this causal category represents a small share of the deaths in both cohorts.

Large differences in period-end survival between the K-M and competing risk curves for some causes of death may seem like evidence of strong interference all on their own. However, it should be noted that in this study the data are extremely thin at the extremes of follow-up, which makes the estimated survival unstable near the end of the study period. The comparison of AUCs mitigates this problem by taking account of survival over the entire follow-up period. Small differences in AUC by cause are reflective of large amounts of congruence between the K-M and competing risk curves across most of the study period.

Based on the results reported here, we again fail to find evidence sufficient to conclude that historical doses of space radiation pose an excess mortality risk for astronauts and cosmonauts. However, it is important to note that future missions of deep space exploration will likely offer much greater doses of space radiation than have historical doses, which will lead to a different risk profile for future astronauts and cosmonauts. In the years to come it is imperative that epidemiologists continue to surveil the astronaut and cosmonaut populations for potential harmful effects of exposure to space radiation, using methods both novel and familiar. Doing so will be integral to supporting human ambitions for further exploration and eventual colonization of our solar system.

## Methods

### Study population

The study population consisted of all NASA astronauts selected since 1959 and all Soviet or Russian cosmonauts selected since 1961 who had been to space at least once before the end of the follow-up period. Follow-up for members of both cohorts began with the beginning of the first spaceflight and ended with either death or the study close date. For astronauts the study close date was 31 July 2018, while for cosmonauts it was 31 December 2017.

The astronaut data used here were taken from official biographies of astronauts maintained on the NASA website, and from information contained in the Astronaut Fact Book^12,13^. The data have been described and analyzed in previous studies of astronaut mortality^6,14^. In prior research, the number of deaths and follow-up time were found to exactly match those of an earlier NASA publication^15^, and subsequent research published by NASA has used the same sources for cohort enumeration^16^.

Data on Soviet and Russian cosmonauts were collected from the Russian-language websites of the Russian Federal Space Agency and the Yuri Gagarin Research & Test Cosmonaut Center, and have also been described previously^17,18^. This database has produced results consistent with other publications of cosmonaut mortality using independently extracted data^6,19^.

This study was exempt from institutional review as the data are publicly available information collected from the internet, and we made no attempt to contact the astronauts or cosmonauts under study or their families.

### Statistical methods

We computed cause-specific survival curves using two different techniques. For each cause-of-death group we first computed standard Kaplan-Meier (K-M) survival curves, treating deaths due to competing causes (any cause other than the cause of interest) as censored events. We then computed survival using competing risks methodology that properly account for all competing causes of death^10^. We compared the two sets of curves for each outcome within each cohort to determine if there was a substantial difference between the survival curves computed using the two methods, which would be expected if a common exposure (e.g. space radiation) were impacting both the cause of death in question and competing causes (thus making the assumption of uninformative censoring inherent in the K-M method false, and biasing the resulting K-M survival curve).

As a quantitative measure of the difference between the naïve K-M curve and the competing-risks curve for each cause of death (i.e. of the bias between the two curves within each outcome), we calculated the AUC for each curve and for each outcome, within cohorts. We then computed both the ratio and the difference in AUC between the K-M and competing risk curves for each cause and for astronauts and cosmonauts separately. These quantities allow for comparison of the total survival experience implied by the two curves. The difference between the areas can be interpreted as the difference in average life-years lived during the study period (in this case from the first space flight until either the end of the period or death); the ratio of the areas is the ratio of those averages. All astronaut analyses were completed using the R statistical software program, with the “survival” package^20,21^. Cosmonaut analyses were completed using a combination of the IBM SPSS software package^22^ and MS Excel, then visualized using R.
